# Clinical Functional Seizure Score (CFSS): a simple algorithm for clinicians to suspect functional seizures

**DOI:** 10.3389/fneur.2023.1295266

**Published:** 2023-11-29

**Authors:** Mohammad Dashtkoohi, Sakineh Ranji-Bourachaloo, Rozhina Pouremamali, Mohadese Dashtkoohi, Raha Zamani, Aysan Moeinafshar, Arshia Shizarpour, Shirin Shakiba, Mohammadali Babaee, Abbas Tafakhori

**Affiliations:** ^1^Students' Scientific Research Center (SSRC), Tehran University of Medical Sciences, Tehran, Iran; ^2^Iranian Center of Neurological Research, Neuroscience Institute, Tehran University of Medical Sciences, Tehran, Iran; ^3^Department of Neurology, Tehran University of Medical Sciences, Tehran, Iran

**Keywords:** seizures, epilepsy, psychogenic nonepileptic seizures, neurobehavioral manifestations, diagnosis

## Abstract

**Purpose:**

Distinguishing functional seizures (FS) from epileptic seizures (ES) poses a challenge due to similar clinical manifestations. The creation of a clinical scoring system that assists in accurately diagnosing patients with FS would be a valuable contribution to medical practice. This score has the potential to enhance clinical decision-making and facilitate prompt diagnosis of patients with FS.

**Methods:**

Participants who met the inclusion criteria were randomly divided into three distinct groups: training, validation, and test cohorts. Demographic and semiological variables were analyzed in the training cohort by univariate analyses. Variables that showed a significant difference between FS and ES were then further scrutinized in two multivariate logistic regression models. The CFSS was developed based on the odds ratio of the discriminating variables. Using the validation group, the optimal cutoff value was determined based on the AUC, and then the CFSS was evaluated in the test cohort to assess its performance.

**Results:**

The developed score yielded an AUC of 0.78 in the validation cohort, and a cutoff point of 6 was established with a focus on maximizing sensitivity without significantly compromising specificity. The score was then applied in the test cohort, where it achieved a sensitivity of 86.96% and a specificity of 73.81%.

**Conclusion:**

We have developed a new tool that shows promising results in identifying patients suspicious of FS. With further analysis through prospective studies, this innovative, simple tool can be integrated into the diagnostic process of FS.

## 1 Introduction

Functional seizures (FS) or psychogenic non-epileptic seizures are characterized by paroxysmal and involuntary movements, sensations, and experiences that resemble epileptic seizures (ES) without associated epileptic changes in the electroencephalogram (EEG) (1). Approximately 20–50% of patients admitted to seizure monitoring units were eventually diagnosed with FS (2–4). Distinguishing between FS and ES can be challenging, especially when both occur simultaneously in ~13% of patients (5). Consequently, diagnosing FS can take anywhere from 1 to 16 years due to the complexity of the situation (6, 7). It is important to understand that anti-seizure medications (ASMs) do not cure or ease the symptoms of FS. They can potentially worsen the condition and bring about more frequent FS episodes. Furthermore, medication side effects and unnecessary hospitalization costs will impose a considerable burden on patients and the healthcare system (4, 7–9). The minimum evidence for diagnosing FS was established in an ILAE special report by LaFrance et al. The report has categorized the diagnosis confidence into four levels: possible, probable, clinically established, and documented. However, the report also acknowledges that, in some instances, access to specialized equipment such as a video electroencephalogram (VEEG) or a neurologist could be limited. In such cases, auxiliary tools can be utilized to aid in the better triage and diagnosis of FS (10). Various approaches, such as checking prolactin levels (11) or innovative methods such as videotaping the episode in patients with motor-type seizures, have been employed to help differentiate FS from ES (12). One way to assist diagnosis is through the use of scoring systems and medical decision-support techniques. Kerr et al. created a scoring system called the dissociative seizure probability score (DSLS), which considers peristaltic behavior, comorbidities, medications, and historical factors in 20 key questions (13). Lenio et al. attempted to validate the DSLS by adding nine additional factors to the original scoring system, resulting in the UC-DSLS. Both scoring systems showed comparable and robust performance (14). Another scoring system developed by Baroni et al. is the Suspected Non-Epileptic Seizure Scale (SS-PNES), which includes 15 questions (15). Despite showing good results, the use of such scoring systems is still limited. We hypothesize that their complexity and multiple variables hinder their widespread use. As a solution, we aimed to develop a simple clinical decision support scoring system. This system can be useful for diagnosing and referring patients with suspected FS as an aid besides clinical suspicion.

## 2 Materials and methods

### 2.1 Patients and variables

In this retrospective cohort study, all patients admitted to the epilepsy monitoring unit of Imam Khomeini Hospital, Tehran, Iran between 4 July 2018 and 20 April 2023 were initially recruited. To ascertain the presence of either FS, ES, or a mixed disorder, two experienced epileptologists independently reviewed the history, physical examinations, and VEEG results and made a final diagnosis. Any disagreement was resolved through consensus, and the patient was only included upon the agreement of both experts.

Patients were included if they met all of the following criteria: 1. admitted to the hospital with suspected seizure disorders that could not be explained by systemic disease, 2. had at least one documented event during VEEG monitoring, and 3. had a comprehensive and reliable interview about the patient's medical history and demographics. Infants and inconclusive diagnoses were excluded. The cohort was divided into three groups: ES, FS, and mixed. The latter consists of patients who have experienced at least one FS event during their admission, as well as at least one ES event caught during admission or had a documented epileptic seizure with VEEG monitoring. Due to the higher prevalence of ES, the selection period for ES patients was narrowed. Patients who were admitted between 21 March 2022 and 20 April 2023 were included in the study. Patients were allocated randomly to three different cohorts: the training cohort, the validation cohort, and the test cohort. The ratio of allocation was 70:15:15, respectively.

We used two categories of variables to analyze patient data: demographics and events. For event-related variables, we selected those that do not require medical expertise to interpret. Therefore, we avoided using semiological categories that are incomprehensible to patients or their companions. Instead, we included general variables such as post-seizure turbulence, repetitive movement, the presence or absence of a trigger, and the duration of the seizure. The patient's demographic details and accounts of the event(s), as reported by the patient and/or caregiver, were procured from the neurologist's notes during the visit preceding admission, while the clinical variables were sourced from the VEEG study reports. Analyzed variables and their definitions are present in the Supplementary material Section 1. This study was approved by the ethical review board of the Tehran University of Medical Sciences, following the Declaration of Helsinki.

### 2.2 Statistical analysis and score development

In the training cohort, demographic and semiological variables were analyzed separately. Significant associations were identified through univariate analyses and then entered into two distinct multivariate logistic regression models to assess independence. Scoring systems were developed based on the odds ratio (OR) of the independent significant predictors. The statistical analyses were conducted using R version 4.1.2. We used a significance level of < 0.05 to determine statistical significance. For univariate analyses, we used the chi-squared test, and for multivariate analyses, we used a logistic regression model. To determine cutoff points for continuous variables, we used the ROC curve and identified the optimal Youden index in the development cohort. We then plotted a ROC curve to determine the most effective cutoff point for the developed score system, prioritizing sensitivity without compromising specificity. In the test cohort, we evaluated the scoring system's diagnostic power using various parameters, including sensitivity, specificity, positive predictive value (PPV), negative predictive value (NPV), and accuracy. We also compared the performance of our developed method to DSLS and reported the results to the test cohort. The formulas that were applied are shown below:


$$\begin{array}{l} Youden\;Index=Sensitivity+Specificity-1\\ Sensitivity\;=\frac{True\;Positives}{True\;Positives+False\;Negatives}\\ Specificity=\frac{True\;Negatives}{True\;Negatives+False\;Positives}\\ Accuracy=\frac{True\;Positives+True\;Negatives}{Total\;Predictions}\\ NPV=\frac{True\;Negatives}{True\;Negatives+False\;Negatives}\\ PPV=\frac{True\;Positives}{True\;Positives+False\;Positives} \end{array}$$

## 3 Results

**Figure 1:**
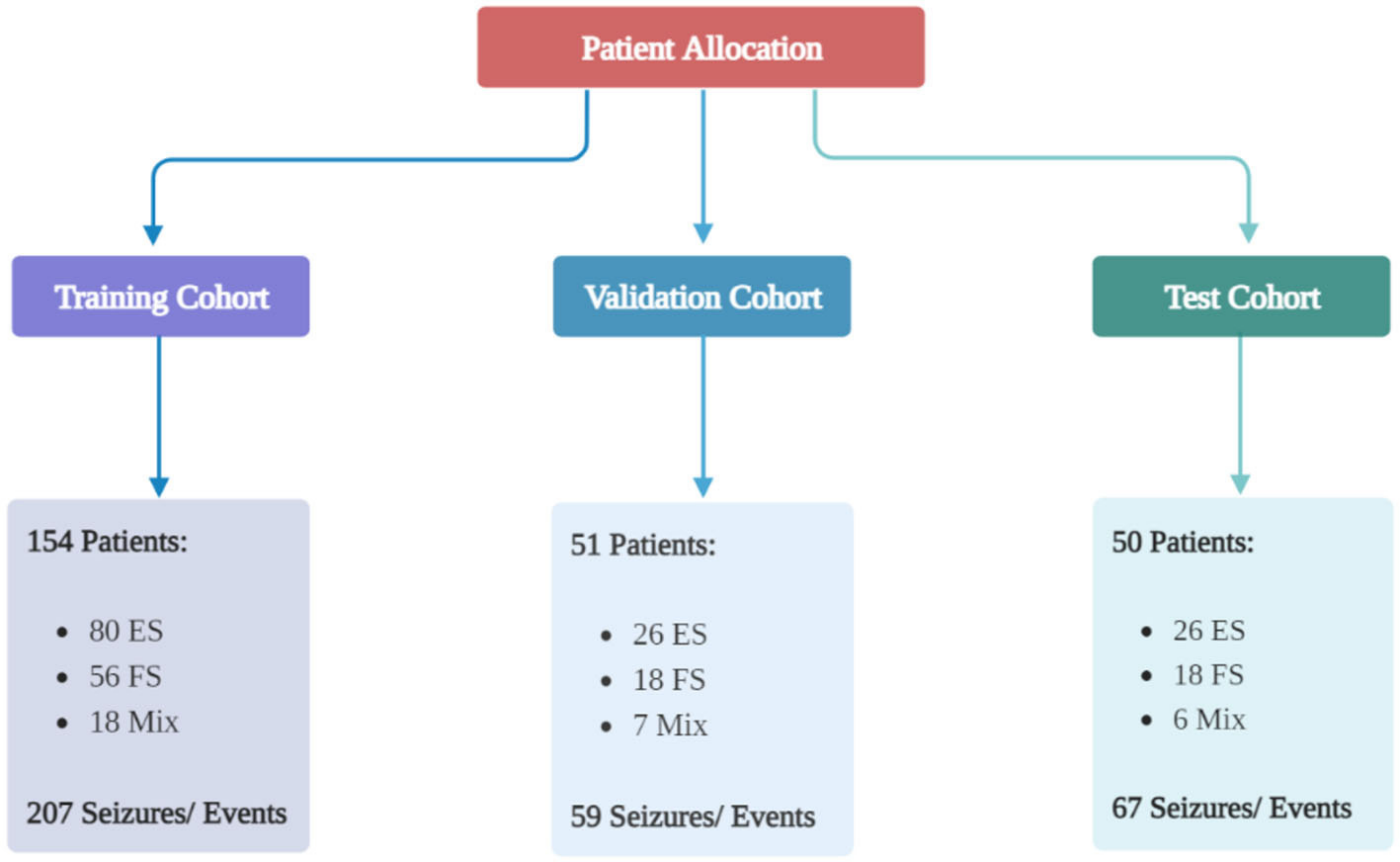
Patient allocation to each group. The patients were divided into three groups randomly: a training group, a validation group, and a test group. The ratio was 70:15:15. Created with BioRender.com.

**Figure 2:**
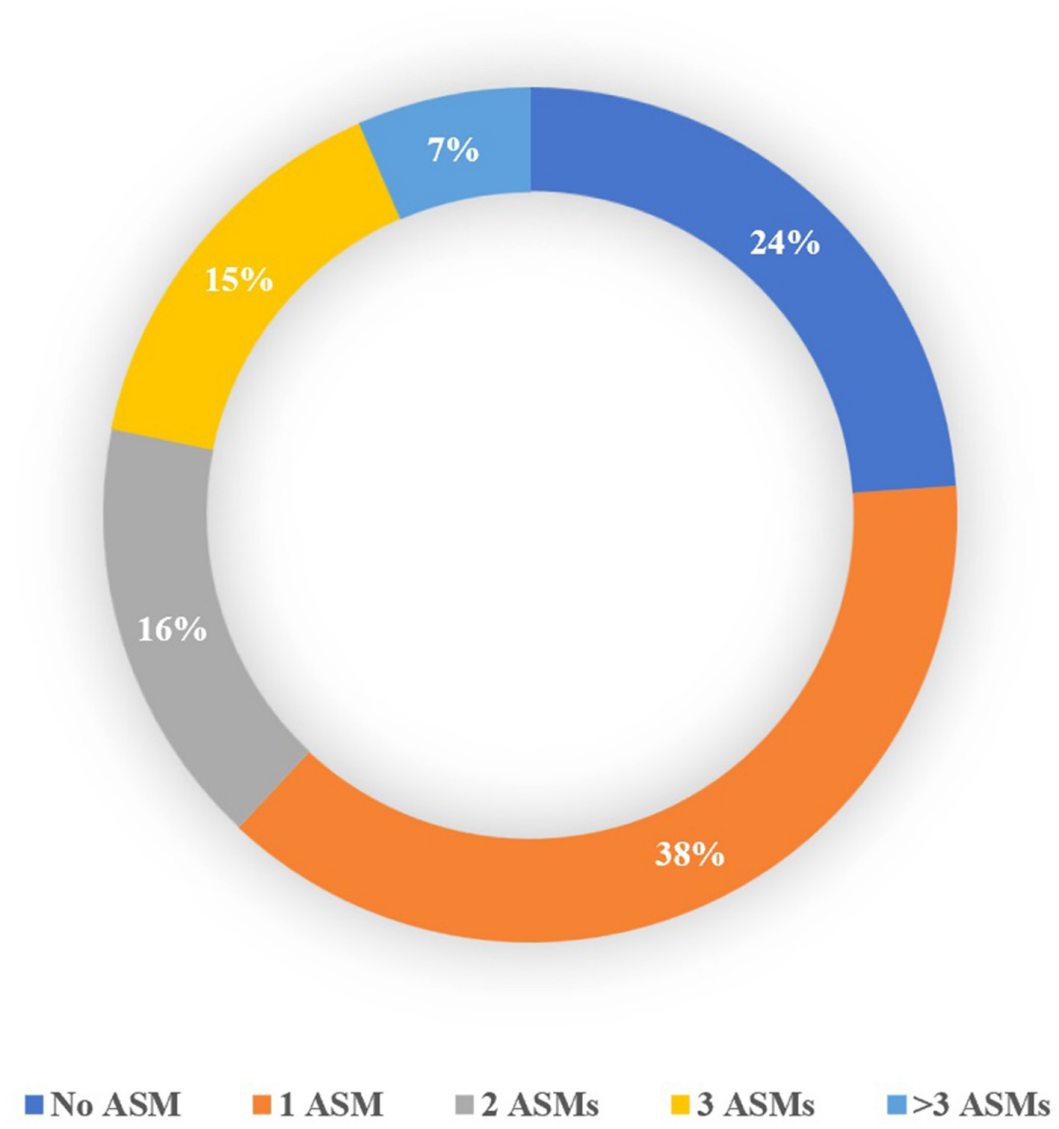
Percentage of patients with FS based on the number of ASMs received before admission to the ward.

The study included 255 patients and 333 seizures/events. Details regarding the type of seizure in patients are available in the Supplementary material Section 2. Patient allocation to each cohort is shown in Figure 1, and Table 1 presents the baseline characteristics of the patients along with the corresponding number of seizures/events in each group. As depicted in Figure 2, a vast majority of patients diagnosed with FS were administered at least one ASM, with only a small percentage of them not receiving any such medication, i.e., 23.81%.

**Table 1:** Characteristics of patients and events in the training, validation, and test cohorts.

	**Training cohort**				**Validation cohort**				**Test cohort**			
	**ES**	**FS**	**Mix**	**Total**	**ES**	**FS**	**Mix**	**Total**	**ES**	**FS**	**Mix**	**Total**
Number of Patients	80	56	18	154	26	18	7	51	26	18	6	50
Female (%)	35 (44.30)	38 (66.67)	13 (72.22)	86 (55.84)	10 (38.46)	17 (94.44)	6 (85.71)	33 (64.71)	16 (61.54)	9 (50.00)	3 (50.00)	28 (56.00)
Age means (SD)	28.17 (13.02)	31.97 (12.74)	30.72 (8.07)	29.88 (12.49)	27.92 (15.44)	35.78 (13.33)	25.00 (8.56)	30.29 (14.36)	25.17 (14.90)	33.00 (18.30)	26.50 (6.47)	28.15 (15.73)
Total events	-	-	-	207	-	-	-	59	-	-	-	67
FS	0	60	19	79	0	18	8	26	0	19	4	23
ES	124	0	4	128	33	0	0	33	42	0	2	44

The AUC values for age at admission, age at the onset of events, and event duration were 0.56, 0.72, and 0.84, respectively. To obtain the highest possible Youden index, we selected cutoff values of ≥19, ≥11, and ≥2 min for age at admission, age at onset, and event duration, respectively. The duration cutoff was rounded to the nearest minute.

Demographic variables showed a significant difference between documented ES and FS patients in terms of age at admission, age at onset of events, gender, marital status, and psychological comorbidities (including major depressive disorder, bipolar disorder, anxiety disorder, and obsessive-compulsive disorder). Among the event features, duration, presence of aura, the occurrence of an event during sleep, post-ictal turbulence (including confusion, nose wiping, Todd's paresis, and aphasia), talking during events, the evolution (changes in the character of seizure in each event), development of repetitive movement (including hyper-motor, jerk, myoclonus, clonic, side-to-side movements, and pelvic thrust), motionlessly lying in the bed after seizure, and rigidity (including tonic, stiffness, spasm, and dystonia) showed a significant difference. Multivariate regression of demographic characteristics revealed that female sex, age at onset ≥11 years, and psychological comorbidities were independent diagnostic features for FS. Duration, evolution, and repetitive movements were variables related to event characteristics that were in favor of FS. Table 2 shows the detailed results of the univariate analyses and multivariate logistic regressions.

**Table 2:** Univariate and multivariate analyses of variables in ES and FS groups.

	**Univariate**				**Multivariate**
**Patients**	**ES (*****n*** = **80)**	**FS/Mix (*****n*** = **74)**	***p-*** **value**	**OR (95%CI)**	***p*** **-value**
Age at admission ≥19 %	74.68	87.83	0.042^*^	1.24 (0.44, 3.57)	0.689
Age at onset of events ≥11 %	48.65	80.88	< 0.001^*^	2.49 (1.16, 5.49)	0.021^*^
Female sex %	45.00	67.57	0.006^*^	2.48 (1.21, 5.24)	0.015^*^
Married %	31.25	51.35	0.014^*^	2.13 (0.95, 4.88)	0.069
Febrile seizure history %	20	9.46	0.074	-	-
Head trauma %	31.25	21.62	0.204	-	-
Positive seizure family history %	29.11	29.73	1	-	-
Psychological comorbidity %	20.00	48.65	< 0.001^*^	3.05 (1.38, 6.95)	0.007^*^
Smoking %	3.75	10.81	0.120	-	-
**Events**	**ES (*****n*** = **128)**	**FS (*****n*** = **79)**	***p-*** **value**	**OR (95%CI)**	***p*** **-value**
Event duration≥ 2 min %	30.00	66.67	< 0.001^*^	4.68 (2.04, 11.27)	< 0.001^*^
Aura %	64.29	39.24	0.001^*^	0.54 (0.23, 1.24)	0.738
Trigger(s) %	4.76	12.31	0.13	-	-
Events in sleep %	23.81	7.59	0.003^*^	0.40 (0.10, 1.36)	0.163
Postictal turbulence %	27.43	12.86	0.027^*^	0.40 (0.14, 1.06)	0.075
Talking %	11.38	23.68	0.029^*^	2.55 (0.83, 8.13)	0.103
Screaming %	2.38	6.41	0.264	-	-
Evolution %	70.40	36.36	< 0.001^*^	0.28 (0.10, 0.75)	0.014^*^
Repetitive movement %	46.09	67.09	0.004^*^	3.87 (1.53, 10.69)	0.005^*^
Motionless lying in bed %	21.43	6.49	0.005^*^	0.39 (0.09, 1.36)	0.159
Behavioral arrest %	46.09	25.32	0.003^*^	0.66 (0.26, 1.62)	0.361
Rigidity %	51.56	32.91	0.010^*^	0.42 (0.16, 1.05)	0.067
Emotional disturbance %	4.69	8.86	0.250	-	-

ES, epileptic seizure; FS, functional seizure; OR, odds ratio; IQR interquartile range; SD, standard deviation. ^*^ denotes statistical significance.

The scores for each variable can be found in Table 3.

**Table 3:** Developed scoring system variables and scores.

	**OR**	**Score**
**Demographic variables**	
Age at onset of events ≥11	2.49	2.5
Female sex	2.48	2.5
Psychological comorbidity	3.05	3
**Event variables**	
Event duration≥ 2 min	4.68	4.5
Evolution	0.28	−3.5
Repetitive movement	3.87	4

**Figure 3:**
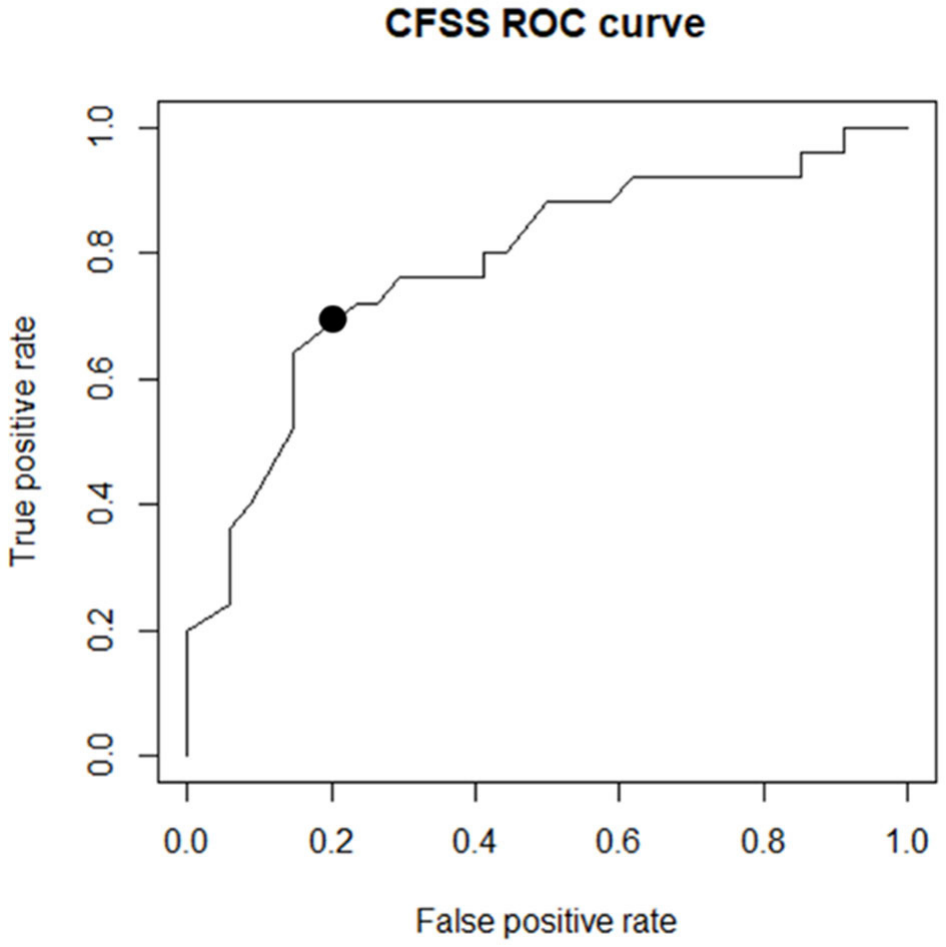
The ROC curve drawn of the scoring system on the validation group. The black point marked in the image shows the determined cutoff.

The generated scoring system showed an AUC of 0.78 in the validation group. To ensure maximum sensitivity in addition to good specificity, a cutoff value of 6 was chosen for the total CFSS score. The ROC curves are shown in Figure 3.

The performance of the scoring system was then evaluated using a cutoff value of 6 in the test group. The CFSS successfully identified 20 FS and 31 ES cases and showed a sensitivity of 86.96% (95%CI: 69.6%−96.6%), a specificity of 73.81% (95%CI: 59.4%−85.4%), and an accuracy of 78.46%. The PPV and NPV of the test were 64.5% (95%CI 47.0%−79.7%) and 91.2% (95%CI 78.7%−97.7%), respectively. Table 4 compares CFSS performance with DSLS results. The results for each event are shown in the Supplementary material Section 3.

**Table 4:** Overall performance of the developed tool (CFSS) and DSLS on the test cohort.

	**True positive**	**False-positive**	**True-negative**	**False-negative**
CFSS	20	11	31	3
DSLS	19	13	29	6

## 4 Discussion

In this study, we developed a novel and simple clinical decision support tool to identify patients with suspected FS. Our developed tool achieved a sensitivity of 86.96% and a specificity of 73.81%. CFSS is based on demographic and semiological factors, including female sex, presence of psychiatric comorbidities (including major depressive disorder, bipolar disorder, anxiety disorders, obsessive-compulsive disorder, and post-traumatic stress disorder), the age at which seizures first occurred (as recalled by the patient) ≥11, the evolution of event (i.e., changes in the characteristics of the seizure during its course), repetitive movement characteristics (including clonus, myoclonus, hyper-motor, tremor, jerky movements, pelvic thrust, and side-to-side movements), and duration of events (how long individual seizures usually last) ≥2 min. CFSS demonstrated superior performance compared to the DSLS scoring system. Distinguishing between FS and ES can be accomplished by considering multiple characteristics, such as seizure characteristics and demographics. Several variables, including neuropsychological impairments, childhood trauma, psychological comorbidities, and frequency of events, can all play a significant role in differentiating between the two types of seizures (2, 16–19). To diagnose FS, we used six differentiating variables.

### 4.1 Duration of events

It is suggested that seizures lasting longer than 2 min are more likely to be FS (16, 20). Seneviratne et al. reported a duration of 123.5 s as an optimal cutoff for recognizing FS (20), which concurs with our findings, which showed a duration longer than 2 min was in favor of FS.

### 4.2 Type of motor phenomena

The motor behavior of psychogenic seizures varies greatly, such that an event may fall on a spectrum from the motor to the non-motor (21). We investigated a variety of motor signs and concluded that the utilization of these characteristics distinguishes ES and FS. A systematic review by Mostacci et al. showed that pelvic thrusting was present in as many as 8–50% of cases with FS among different studies (16). Another study reported that pelvic thrusting was highly specific for FS but was not as sensitive (22). Avbersek et al. described this type of movement as a good sign for distinguishing between FS and ES, with the exception of frontal lobe seizures (23). Side-to-side head movements are also highly specific for FS, particularly when differentiating between FS and generalized tonic-clonic seizure (GTCS) (23, 22). While bilateral head movements are more prevalent in FS, unilateral movements are more common in GTCS and frontal lobe seizures (16). Mostacci et al. reported that 7–76% of patients with FS had episodes of “unresponsiveness without motor symptoms, often accompanied by apparent atonia” (16). In one study, 25% of patients with FS had episodes of falling, which is considered atonia. Nevertheless, we should differentiate this symptom from organic syncope and generalized tonic and atonic seizures (24). Many studies have attributed hypermotor/hyperkinetic movements to FS. Hyperkinetic movements of the limbs are bilateral, asynchronous, and asymmetric (25). In a study comparing temporal lobe seizures and FS, hyperkinetic seizures were more common in the latter (26). Moreover, Groppel et al. observed hyperkinetic movements of the limbs in 14.8% of patients with FS. Trembling was present in 96.3% of their sample (24). Hubsch et al. also reported tremors in 43% of their patients (27). In our research, the term repetitive movements encompasses a range of motor behaviors, including clonus, myoclonus, hyper-motor, tremor, jerky movements, pelvic thrust, and side-to-side movements. The decision to focus on this variable was motivated by two primary factors. First, it seems that patients or their companions often face difficulties in providing an accurate description of the patient's movements (28). Second, the occurrence rate for each movement type in our samples was relatively low. Consequently, we chose to group all the different types of movements under the category of repetitive movements to ensure the accountability of results.

### 4.3 Evolution

In a study conducted by Chen et al., a gradual evolution of the seizure was more prevalent in the ES group, and a cutoff age of 70s was reported for reaching its peak; on the contrary, the FS group had a more abrupt onset. The presumed cutoff had a lower sensitivity in differentiating between FS and frontal lobe seizures since the latter is prone to an abrupt onset as well (29); this was also replicated in other studies (16, 30). However, another study identified an abrupt onset as a highly sensitive sign of ES (22). This is in contrast to our results, where a lack of evolution was associated with FS.

### 4.4 Age at onset

Regarding demographic factors, studies have consistently depicted a younger age of onset in patients with ES (17, 26, 31). Hoepner et al. reported that only 10% of their patients with pure FS had an onset before the age of 15, and when suspecting FS with an onset before this age, clinicians should not overlook a coexisting ES (32). These findings are close to our results, which have a cutoff of 11 years for the age at the onset of events. In contrast to the previous scoring systems, we chose “age at onset” instead of “duration of disease” since it is independent of the time by which patients seek medical help and of the accessibility of seizure monitoring facilities (33).

### 4.5 Female sex and marital status

It has been widely demonstrated that FS is predominant in female sex (26, 31). A meta-analysis conducted by Gilmour et al. concluded that the female sex had the highest sensitivity for FS (17). Hoepner et al. (32) observed that in both the pure FS and mixed groups, patients were mostly female subjects. Nevertheless, a cohort conducted in the UK showed similar rates in the marital state of FS, but the rates were also comparable to the general population. An explanation for this variance might be the socioeconomic and cultural differences between the two populations (34). One study demonstrated no significant difference in the marital status rates of FS and ES patients in Iran; however, both of these groups had lower marriage rates than the general population (34). This is in contrast to our study, as the ES group had significantly lower marriage rates than the FS.

### 4.6 Psychological comorbidity

FS and ES patients are distinct in their psychiatric and traumatic histories. Kerr et al. (31) found that patients with FS had higher rates of stressful life events, including sexual and physical assault, along with higher rates of substance abuse. In a systematic review, psychiatric comorbidities had a prevalence of 53–100% in the FS groups, with depression being the most common, followed by anxiety and posttraumatic stress disorders (PTSDs) (35). One study compared the pure FS and mixed groups and observed no significant variation (36). Another study, in contrast, demonstrated a high prevalence of mood disorders and anxiety in epileptic patients (37). However, in a meta-analysis comparing FS and ES, axis 1 disorders such as depression, anxiety, and PTSD, along with personality disorders, were in favor of FS (35). It is also suggested that cluster B personality disorders are significantly more common in FS, namely, borderline and depressive personality disorders (38). Emotional dysregulation is common in patients with FS and is highly associated with axis 1 disorders, which are also prevalent in them (39). A recent study showed that patients with FS had a higher frequency of childhood trauma compared to ES, regardless of the type of trauma (19). This discrepancy is also replicated in our study, with a 48.65% prevalence of psychological comorbidities in patients with FS compared to a 20.00% prevalence in the ES group.

### 4.7 Comparison to previous scoring systems

Researchers have proposed several scoring systems over the years. Kerr et al. developed a tool addressing peri-ictal behavior, comorbidities and medications, and historical factors. The final scoring system, named dissociative seizure likelihood score (DSLS), consists of 20 key questions. They also compared the relative performance of neurologists, trained premedical students, and a naïve classifier who diagnoses all patients as having ES (13). Lenio et al. attempted to validate DSLS, added nine other factors to the original scoring system (UC-DSLS), and applied both to their sample. The overall performance of both of them was comparable and robust (14). Baroni et al. developed the Scale for Suspicion of Psychogenic Non-epileptic Seizures (SS-PNES), which included 15 questions. Initially, they conducted a systematic review, identified 49 discriminatory factors, added 2 other factors based on expert opinion, and prospectively evaluated them afterward (15). Trainor et al. administered seven neuropsychological and neuropsychiatric questionnaires to the patients and included the top 20 discriminatory items in the Anxiety, Abuse, and Somatization Questionnaire (AASQ) (40). Reuber et al., Chen et al., and Wardrope et al., developed two sets of questionnaires [paroxysmal event profile (PEP) and paroxysmal event observer (PEO)] in a retrospective manner. They recruited patients with a transient loss of consciousness (TLOC) who were diagnosed with either FS, ES, or syncope and administered these questionnaires to the patients and a witness. The items included in PEP and PEO were based on previous research (41, 42) and expert opinion, and a neurologist made the discrimination between ES and FS based on VEEG findings. At last, they concluded that, although both PEP and PEO are useful discriminatory tools, their combination is more powerful in distinguishing between ES, FS, and syncope (43–45). Our study demonstrated that the CFSS outperformed the DSLS in the test cohort. CFSS achieved comparable accuracy with previous tools, with a considerably lower number of items, which increased the ease of utilization.

### 4.8 Limitations

This study has a few limitations. First, we obtained data on event duration, evolution, and repetitive movement from inpatient records rather than directly from patients or caregivers. Consequently, the accuracy of our scale may vary when assessing patients based on real-time interviews in a clinical setting. To address these concerns, future studies should adopt a prospective approach to further validate CFSS. Moreover, to utilize this tool in low-resource settings, the performance of general practitioners or other healthcare providers without neurologic expertise requires evaluation. Additionally, our sample size was small, and we only utilized data from one tertiary center; therefore, larger studies are necessary to ensure broader applicability. Unfortunately, due to insufficient data, we were unable to measure interrater variability and analyze motor presentations and psychological comorbidity variables in greater detail. However, examining these variables more closely could potentially improve differentiation.

## 5 Conclusion

Despite the use of various scoring systems and questionnaires, a significant majority (76%) of patients with pure FS at our center were taking ASM. This raises concerns about the effectiveness of current scoring systems in detecting such patients. Herein, we developed the CFSS, a more straightforward and practical decision support tool that can assist physicians in identifying patients with FS. The CFSS showed comparable accuracy to prior tools while using fewer variables. The CFSS has the potential to be a part of clinical practice, which is especially significant in countries with limited access to epileptologists and VEEG units.

## Data availability statement

The raw data supporting the conclusions of this article will be made available by the authors, without undue reservation.

## Ethics statement

This study was approved by the Ethical Review Board of Tehran University of Medical Sciences. All steps of the study are conducted in accordance with the local legislation and institutional requirements. Written informed consent for participation was not required from the participants or the participants' legal guardians/next of kin in accordance with the national legislation and institutional requirements.

## Author contributions

MohamD: Conceptualization, Formal analysis, Methodology, Project administration, Visualization, Writing – original draft, Writing – review & editing. SR-B: Data curation, Resources, Supervision, Writing – review & editing. RP: Data curation, Investigation, Writing – original draft. MohadD: Data curation, Investigation, Writing – original draft. RZ: Data curation, Formal analysis, Writing – original draft. AM: Data curation, Investigation, Writing – review & editing. AS: Data curation, Investigation, Writing – review & editing. SS: Investigation, Resources, Writing – review & editing. MB: Data curation, Resources, Writing – review & editing. AT: Conceptualization, Resources, Supervision, Validation, Writing – review & editing.
