# Supplementary material for: Clinical Functional Seizure Score (CFSS): a simple algorithm for clinicians to suspect functional seizures
Source: Front Neurol. 2023 Nov 29;14:1295266. doi: 10.3389/fneur.2023.1295266 (PMC10716468; doi:10.3389/fneur.2023.1295266)
Supplement: Supplementary file 1 [file Table_1.DOCX]

| Variable | Definition |
| --- | --- |
| Age at admission | Age of the patient when they are admitted to epilepsy monitoring unit. |
| Age at Onset of Events | Patient recalls their first seizures/events at that age. |
| Sex | Individual's biological classification as male or female. |
| Marital status | Whether an individual is married or not. |
| Febrile Seizure History | Whether an individual has experienced seizures triggered by fever in the childhood. |
| Head Trauma | Whether an individual has experienced any traumatic brain injuries in the past. |
| Positive Seizure Family History | Whether there is a history of seizures in the patient's first- or second-degree family. |
| Psychological Comorbidity | Co-existing psychological conditions that a patient may have alongside their seizure disorder including major depressive disorder, bipolar disorder, anxiety disorders, obsessive-compulsive disorder and post-traumatic stress disorder. (based on patient or caregiver declaration) |
| Smoking | Whether an individual smokes tobacco product. |
| Event Duration | How long individual seizures usually last |
| Aura | Sensations that a seizure/ event is about to occur. |
| Trigger(s) | Whether there are factors that may induce seizures/ events in an individual. |
| Events in Sleep | Whether patient experiences seizures during sleep |
| Postictal Turbulence | Todd's paresis, coughing, nose wiping, aphasia, and confusion all are considered as postictal turbulence, motionless lying in bed, behavioral arrest |
| Talking | Talking during events including communicating, non-communicating, grunting, or vocalizing types |
| Screaming | To utter a long loud piercing sound during the seizures/ events |
| Evolution | The changes in the characteristics of the seizure during its course. |
| Repetitive Movement | Including clonus, myoclonus, hyper-motor, tremor, jerky movements, pelvic thrust, and side-to-side movements. |
| Motionless lying in bed* | Post ictal hypokinetic status or absolutely still |
| Behavioral Arrest* | The non-responsiveness during seizures/ events |
| Rigidity | Including movements that resemble dystonia, tonic spasm or stiffness during seizures/ events |

Section 1:

The analyzed variables and their definitions are presented below. *These two variables are analyzed once in the postictal turbulence variable and once separately as more specific entities.

Section 2:

| Case | Mix | Focal/ Generalized |
| --- | --- | --- |
| 1 |  | Focal epilepsy with left central origin |
| 2 |  | Focal epilepsy with mesial temporal origin. |
| 3 |  | Focal seizure with right frontal and left temporal origin |
| 4 |  | Focal epilepsy with mesial temporal origin. |
| 5 |  | Genetic generalized epilepsy (IGE) |
| 6 |  | Focal epilepsy with multifocal origins. |
| 7 |  | Focal epilepsy with occipital lobe origin (L>R) |
| 8 |  | Focal epilepsy with right frontal origine. |
| 9 |  | Focal epilepsy with right mesial temporal origin |
| 10 |  | Focal epilepsy with left posterior temporal origin |
| 11 |  | Focal epilepsy with occipital part of right fusiform gyrus. |
| 12 |  | Focal epilepsy with left fronto-temporal origin. |
| 13 |  | Focal epilepsy with left parietal origin |
| 14 |  | Genetic generalized epilepsy |
| 15 |  | Focal epilepsy with occipital lobe origin (L>R) |
| 16 |  | Focal epilepsy with right mesial temporal origin |
| 17 |  | Focal epilepsy at left side mainly left fronto-central region. |
| 18 |  | Focal epilepsy with right (lateral) temporal origin |
| 19 |  | Genetic generalized epilepsy |
| 20 |  | Focal epilepsy with probable left temporal origin |
| 21 |  | Focal epilepsy with left fronto-temporal origin. opercular/insular is the most probable origin. |
| 22 |  | Focal epilepsy with right mesial temporal origin |
| 23 |  | Progressive myoclonus epilepsy |
| 24 |  | Focal epilepsy with mesial temporal origin |
| 25 |  | Focal epilepsy with left mesial temporal origin |
| 26 |  | Generalized epileptic encephalopathy. |
| 27 |  | Focal epilepsy with right mesial temporal origin. |
| 28 |  | Focal epilepsy with multifocal origins. |
| 29 |  | Focal epilepsy probably with mesial frontal origin (R>L) |
| 30 |  | Genetic generalized epilepsy (IGE). |
| 31 |  | epileptic encephalopathy with mixed generalized and multifocal foci (bi-temporal, lt frontal regions) |
| 32 |  | Focal epilepsy with left parieto-occipital origin |
| 33 |  | Focal epilepsy with left mesial frontal origin (left SSMA) |
| 34 |  | Focal epilepsy with multifocal origins. |
| 35 |  | Epileptic encephalopathy like Lennox-Gastaut syndrome |
| 36 |  | Focal epilepsy with right mesial temporal origin |
| 37 |  | Focal epilepsy with mesial temporal origin. |
| 38 |  | Focal epilepsy with right lateral frontal (FEF) origin |
| 39 |  | Epileptic encephalopathy with mixed generalized and focal seizures |
| 40 |  | Focal epilepsy with right temporal origin |
| 41 |  | Focal epilepsy with bilateral mesial temporal origin |
| 42 |  | Focal epilepsy with right temporal origin. |
| 43 |  | Focal epilepsy with left mesial temporal origin |
| 44 |  | Focal epilepsy with left hemisphere involvement |
| 45 |  | Epileptic encephalopathy with mixed focal and generalized seizures like Lennox-Gastaut syndrome |
| 46 |  | Genetic generalized epilepsy |
| 47 |  | Focal epilepsy with left mesial temporal origin. |
| 48 |  | Focal epilepsy with right temporal origin (mesial>lateral) |
| 49 |  | Focal epilepsy with right mesial temporal origin |
| 50 |  | Focal epilepsy with left fronto-central origin |
| 51 |  | Focal epilepsy with two origins in right anterior temporal and left temporo-occipital regions |
| 52 |  | Focal epilepsy with most probable right temporo-occipital origin. |
| 53 |  | Mixed generalized and focal epilepsy. |
| 54 |  | Focal epilepsy mesial frontal origin |
| 55 |  | Focal epilepsy with right mesial temporal origin |
| 56 |  | Genetic generalized epilepsy. |
| 57 |  | Genetic generalized epilepsy. |
| 58 |  | Focal epilepsy with Lt Ant temporal origin. |
| 59 |  | Focal epilepsy with left central origin |
| 60 |  | Focal epilepsy with Lt Ant temporal origin |
| 61 |  | Focal epilepsy with right frontal lobe origin |
| 62 |  | Focal epilepsy. For precise localization and decide on epilepsy surgery invasive video-EEG monitoring and perimetry is recommended. |
| 63 |  | Focal epilepsy with left mesial temporal origin. |
| 64 |  | Focal epilepsy with right fronto-central origin |
| 65 |  | Focal epilepsy with left mesial temporal origin. |
| 66 |  | Genetic generalized epilepsy |
| 67 |  | Focal epilepsy with right temporal origin |
| 68 |  | Focal seizure with right frontal and left temporal origin |
| 69 |  | Focal epilepsy with left SMA origin |
| 70 | Mix | Mix (genetic generalized epilepsy). Recent attacks of tonic and myoclonic events are in favor of FS. |
| 71 |  | Focal epilepsy with right occipital lobe origin |
| 72 |  | Focal epilepsy with left temporo-occipital origin |
| 73 |  | Generalized epileptic encephalopathy with reflex subcortical myoclonus |
| 74 |  | Genetic generalized epilepsy |
| 75 |  | Progressive myoclonic epilepsy |
| 76 | Mix | Focal epilepsy with left temporal origin and FS |
| 77 |  | Focal epilepsy with bi-temporal origin (lateral temporal R>L). |
| 78 |  | Focal epilepsy with left mesial temporal origin |
| 79 |  | Focal epilepsy with left frontal operculum or insula and less probably left mesial frontal origin should be considered. |
| 80 | Mix | Mixed focal epilepsy with right frontal origin and FS |
| 81 |  | Genetic generalized epilepsy |
| 82 |  | Genetic generalized epilepsy |
| 83 |  | Lance – Adams syndrome |
| 84 |  | Focal epilepsy with left mesial temporal origin |
| 85 |  | Idiopathic generalized epilepsy |
| 86 |  | Focal epilepsy with right lateral temporal origin |
| 87 |  | Focal epilepsy with left temporal origin (L>M). |
| 88 |  | Focal epilepsy with right temporal origin (L>M) |
| 89 |  | Focal epilepsy with right temporal lobe origine |
| 90 |  | Mixed epileptic encephalopathy most probably Lennox-Gastaut |
| 91 |  | Focal epilepsy with right temporo-occipital origin |
| 92 |  | Genetic generalized epilepsy |
| 93 |  | Focal epilepsy with right temporal origin |
| 94 |  | Focal epilepsy with two foci in right mesial temporal and left posterior temporal regions |
| 95 |  | Focal epilepsy with right mesial temporal origin |
| 96 |  | Focal epilepsy with right mesial temporal origin |
| 97 |  | Frontal lobe epilepsy particularly left mesial frontal epilepsy should be considered |
| 98 |  | Generalized epileptic encephalopathy |
| 99 | Mix | Focal epilepsy with left temporal origin and FS |
| 100 |  | Focal epilepsy with involvement of lateral and mesial temporal |
| 101 |  | Lennox-Gastaut syndrome |
| 102 |  | Focal epilepsy in right lateral temporal region |
| 103 |  | Focal epilepsy with left mesial temporal origin. |
| 104 |  | Focal epilepsy with left central origin. |
| 105 |  | Genetic generalized epilepsy |
| 106 | Mix | Mixed genetic generalized epilepsy and FS |
| 107 |  | Focal epilepsy with right mesial temporal origin. |
| 108 |  | Idiopathic generalized epilepsy |
| 109 |  | Focal epilepsy with left anterior temporal |
| 110 |  | Focal epilepsy with left mesial temporal origin |
| 111 |  | Focal epilepsy with left mesial temporal origin |
| 112 |  | Focal epilepsy with mesial temporal origin |
| 113 |  | Generalized epilepsy |
| 114 |  | Mixed focal and generalized epileptic encephalopathy. |
| 115 |  | Focal epilepsy with left mesial temporal origin |
| 116 |  | Focal epilepsy with left mesial temporal origin. |
| 117 | Mix | Mixed focal epilepsy with right mesial temporal and FC |
| 118 |  | Focal epilepsy probably with mesial frontal origin (R>L). |
| 119 |  | Multifocal with left anterior temporal origin |
| 120 |  | Focal epilepsy with mesial temporal origin |
| 121 | Mix | Focal epilepsy with mesial temporal origin and FC |
| 122 |  | Mixed focal and generalized epileptic encephalopathy |
| 123 |  | Focal epilepsy with mesial temporal origin |
| 124 |  | Focal epilepsy with mesial temporal origin |
| 125 |  | Focal epilepsy with mesial temporal origin |
| 126 |  | Focal epilepsy in right mesial temporal |
| 127 |  | Focal epilepsy with left mesial temporal origin |
| 128 |  | Focal epilepsy with involvement of lateral and mesial temporal |
| 129 |  | Generalized epilepsy |
| 130 |  | Primary generalized epilepsy |
| 131 |  | Focal epilepsy lateralized to right side |
| 132 |  | Focal epilepsy with mesial temporal origin |
| 133 |  | Idiopathic generalized epilepsy |
| 134 |  | Lennox-gastaut syndrome |
| 135 |  | Left frontal lobe epilepsy |
| 136 |  | Focal epilepsy with mesial temporal origin |
| 137 |  | Primary generalized epilepsy |
| 138 |  | Focal epilepsy with probably right temporal origin |
| 139 |  | Focal epilepsy with mesial temporal origin |
| 140 | Mix | Genetic generalized epilepsy |
| 141 | Mix | Focal epilepsy with left central origin and FS |
| 142 | Mix | Genetic generalized epilepsy and FS |
| 143 | Mix | Focal epilepsy with bi-parietal lobe epilepsy and FS |
| 144 | Mix | Focal epilepsy and FS Epileptogenic lesion was the PVNH and pachygyria of Right temporo-ocipito-insular region. |
| 145 | Mix | Focal epilepsy with origin of right frontal and PNES. |
| 146 | Mix | Mixed focal right parieto-temporal epilepsy and FS |
| 147 | Mix | Focal epilepsy mixed with right mesial temporal origin and FS |
| 148 | Mix | Mixed focal epilepsy with temporo-occipital origin and FS |
| 149 | Mix | Mixed focal epilepsy with right lateral temporal region and FS |
| 150 | Mix | Focal epilepsy with right temporooccipital Epilepsy and FS |
| 151 | Mix | Focal epilepsy with left parietal origin and FS |
| 152 | Mix | Mixed generalized epilepsy and FS |
| 153 | Mix | Mixed focal epilepsy with frontal origin (L>R) and FS |
| 154 | Mix | Focal with right temporo-occipital origin and FS |
| 155 | Mix | Focal with left temporo-occipital origin and FS |
| 156 | Mix | Focal epilepsy with Lateral Anterior temporal origin and FS |
| 157 | Mix | right parietal and the other in left posterior temporal lobe should be considered and FS |
| 158 | Mix | Idiopathic generalized epilepsy and FS |
| 159 | Mix | Focal epilepsy with probable frontal origin and FS |
| 160 | Mix | Focal epilepsy with right frontal origin and FS |
| 161 | Mix | Focal epilepsy with right mesial temporal origin and FS |
| 162 | Mix | Mixed generalized epilepsy and FS |
| 163 | Mix | Focal epilepsy with probably mesial frontal origin and FS |

Section 3:

Event based results of CFSS and DSLS

| Event Number | CFSS | DSLS Probability | Diagnosis |
| --- | --- | --- | --- |
| 1 | FN | 96.9% ES, FN | Mix, FS |
| 2 | TP | 57.2% FS, TP | Mix, FS |
| 3 | TP | 65.7% ES, FN | Mix, FS |
| 4 | FN | 61.7% ES, FN | Mix, FS |
| 5 | TP | 82.4% FS, TP | FS |
| 6 | TP | 80.5% FS, TP | FS |
| 7 | TP | 69.0% FS, TP | FS |
| 8 | TP | 52.3% FS, TP | FS |
| 9 | FN | 52.0% FS, TP | FS |
| 10 | FN | 65.6% ES, FN | FS |
| 11 | TP | 88.2% FS, TP | ES |
| 12 | TP | 75% FS, TP | ES |
| 13 | TP | 94.7% FS, TP | FS |
| 14 | TP | 85.1% FS, TP | FS |
| 15 | FN | 52.0% FS, TP | FS |
| 16 | TP | 89.4 FS, TP | FS |
| 17 | TP | 89.0% FS, TP | FS |
| 18 | TP | 62.0% ES, FN | FS |
| 19 | TP | 82.2% FS, TP | FS |
| 20 | TP | 57.6% ES, FN | FS |
| 21 | TP | 57.3% FS, TP | FS |
| 22 | TP | 66.0% FS, TP | FS |
| 23 | TP | 68.6% FS, TP | FS |
| 24 | TP | 76.4% FS, TP | Mix, FS |
| 25 | TP | 78.6% FS, TP | Mix, FS |
| 26 | TN | 89.4% FS, FP | ES |
| 27 | FP | 92.5% FS, FP | ES |
| 28 | TN | 91.5% FS, FP | ES |
| 29 | FP | 86.4% ES, TN | ES |
| 30 | FP | 67.6% FS, FP | ES |
| 31 | FP | 62.0% FS, FP | ES |
| 32 | FP | 52.5% FS, FP | ES |
| 33 | TN | 52.7% ES, TN | ES |
| 34 | TN | 61.6% ES, TN | ES |
| 35 | TN | 66,9% ES, TN | ES |
| 36 | TN | 77.1% ES, TN | ES |
| 37 | TN | 56.9% ES, TN | ES |
| 38 | TN | 53.3% ES, TN | ES |
| 39 | TN | 63.4% ES, TN | ES |
| 40 | TN | 50.3% FS, FP | ES |
| 41 | TN | 69.6% FS, FP | ES |
| 42 | TN | 64.2% FS, FP | ES |
| 43 | TN | 52.7% ES, TN | ES |
| 44 | FP | 50% ES, TN | ES |
| 45 | FP | 56.2% FS, FP | ES |
| 46 | TN | 86.7% ES, TN | ES |
| 47 | TN | 89.2% ES, TN | ES |
| 48 | TN | 86.6% ES, TN | ES |
| 49 | TN | 84.8% ES, TN | ES |
| 50 | TN | 86.2% ES, TN | ES |
| 51 | FP | 61.5% ES, TN | ES |
| 52 | TN | 58% ES, TN | ES |
| 53 | TN | 67.1% ES, TN | ES |
| 54 | TN | 64.4% ES, TN | ES |
| 55 | TN | 51.1% FS, FP | ES |
| 56 | TN | 67.0% ES, TN | ES |
| 57 | TN | 55.2% ES, TN | ES |
| 58 | FP | 65.3% ES, TN | ES |
| 59 | TN | 64.7% ES, TN | ES |
| 60 | FP | 52.8% FS, FP | ES |
| 61 | TN | 79.0% ES, TN | ES |
| 62 | FP | 82.0% ES, TN | ES |
| 63 | TN | 91.9% ES, TN | ES |
| 64 | TN | 91.8% ES, TN | ES |
| 65 | TN | 81.1% ES, TN | ES |
| 66 | TN | 59.3% FS, FP | ES |
| 67 | TN | 72.6% ES, TN | ES |
